# Multimodal Correlates of Longitudinal Functional Decline in Behavioral Variant Frontotemporal Dementia (bvFTD)

**DOI:** 10.3390/jpm16080415

**Published:** 2026-08-03

**Authors:** Electra Chatzidimitriou, Georgios Ntritsos, Eleni Poptsi, Emmanouil Tsardoulias, Andreas L. Symeonidis, Magda Tsolaki, Panos Charalambous, Chrissa Sioka, Eleni Aretouli, Ioannis Iakovou, Katherine P. Rankin, Panagiotis Ioannidis, Despina Moraitou

**Affiliations:** 1 Department of Cognition, Brain and Behavior, School of Psychology, Faculty of Philosophy, Aristotle University of Thessaloniki (AUTh), 54124 Thessaloniki, Greece; demorait@psy.auth.gr; 2 2nd Department of Neurology, AHEPA University Hospital, Aristotle University of Thessaloniki (AUTh), 54636 Thessaloniki, Greece; ispanagi@auth.gr; 3 Laboratory of Neurodegenerative Diseases, Center for Interdisciplinary Research and Innovation, Aristotle University of Thessaloniki (CIRI-AUTh), 57001 Thessaloniki, Greece; poptsi.e@alzheimer-hellas.gr (E.P.); tsolakim1@gmail.com (M.T.); 4 Department of Economics, School of Economics and Management Sciences, University of Ioannina, 45110 Ioannina, Greece; gntritsos@uoi.gr; 5 Greek Association of Alzheimer’s Disease and Related Disorders (GAADRD), Petrou Sindika 13 Str, 54643 Thessaloniki, Greece; 6 School of Electrical and Computer Engineering, Faculty of Engineering, Aristotle University of Thessaloniki (AUTh), 54124 Thessaloniki, Greece; etsardou@ece.auth.gr (E.T.); asymeon@eng.auth.gr (A.L.S.); 7 2nd Academic Department of Nuclear Medicine, AHEPA University Hospital, Aristotle University of Thessaloniki (AUTh), 54636 Thessaloniki, Greece; charalambous.pa@hotmail.com (P.C.); iiakovou@auth.gr (I.I.); 8 Department of Nuclear Medicine, University Hospital of Ioannina, 45500 Ioannina, Greece; csioka@uoi.gr; 9 Department of Psychology, School of Social Sciences, University of Ioannina, 45110 Ioannina, Greece; earetouli@uoi.gr; 10 The Edward and Pearl Fein Memory and Aging Center, Department of Neurology, University of California, San Francisco, San Francisco, CA 94158, USA; kate.rankin@ucsf.edu

**Keywords:** bvFTD, functional decline, longitudinal study, cognitive deficits, behavioral disturbances, personality changes, neuroimaging biomarkers, SPECT imaging

## Abstract

**Background and Objectives:** Functional decline is a major determinant of disability, caregiver burden, and loss of independence in behavioral variant frontotemporal dementia (bvFTD). Although bvFTD is characterized by early and rapidly progressive deterioration in everyday functioning, the multimodal contributors associated with longitudinal functional change remain insufficiently understood. The present study aimed to identify the strongest cognitive, behavioral, personality, and neuroimaging correlates of longitudinal functional deterioration in bvFTD using an integrated multimodal framework over a 12-month follow-up period. **Methods:** Twenty-seven patients diagnosed with bvFTD were recruited from the 2nd Neurology Clinic of the “AHEPA” University Hospital in Thessaloniki, Greece, and underwent comprehensive face-to-face neuropsychological assessment for the evaluation of a wide range of cognitive domains, alongside caregiver-based evaluations of behavioral disturbances, personality changes, and functional abilities, at baseline, 6 months, and 12 months. Brain perfusion single-photon emission computed tomography (SPECT) was acquired only at baseline, with regional cerebral blood flow (rCBF) quantified using a Brodmann area (BA)-based approach. Functional status was assessed using the Disability Assessment for Dementia (DAD) as the primary outcome, while the Frontotemporal Dementia Rating Scale (FRS) served as a secondary measure. Repeated-measures analysis of variance (ANOVA) was used to characterize longitudinal changes across cognitive, behavioral, personality, and functional domains, while linear mixed-effects (LME) models were applied to identify longitudinal correlates of functional decline and sources of inter-individual variability in functional outcomes. **Results:** Significant progressive decline in functional abilities was observed over the 1-year follow-up period, consistent with an aggressive and rapidly deteriorating clinical course in bvFTD. Reductions in functional performance were evident across both basic and instrumental activities of daily living, with deterioration being more pronounced in instrumental activities. Based on the final LME model, greater apathy-related (negative) behavioral symptoms, global cognitive impairment, attentional and processing speed deficits, and impaired inhibitory control were independently associated with poorer longitudinal functional outcomes (*p* < 0.001). At the neuroimaging level, reduced baseline perfusion in the right BA 24 within the anterior cingulate cortex was also significantly associated with greater loss of functional independence over time (*p* < 0.001). **Conclusions:** Longitudinal functional decline in bvFTD reflects the combined disruption of behavioral regulation, global cognition, executive control, and frontal–cingulate network integrity. The findings provide a preliminary foundation for future research investigating factors associated with accelerated functional decline. Further validation in larger, multicenter longitudinal cohorts is needed to determine the prognostic relevance of these factors and their potential applicability to patient stratification and individualized care planning.

## 1. Introduction

Behavioral variant frontotemporal dementia (bvFTD) is the most common clinical subtype of frontotemporal lobar degeneration (FTLD) and is characterized by early and prominent alterations in personality, social conduct, motivation, and executive control [1,2,3]. In contrast to other neurodegenerative syndromes such as Alzheimer’s disease (AD), where episodic memory impairment is often the dominant early feature, bvFTD is primarily defined by profound changes in behavior and socio-emotional functioning that emerge early in the disease course and substantially compromise patients’ ability to function independently in daily life [1,2,3,4].

A core clinical feature of bvFTD is the marked and relatively early impairment in everyday functional abilities, including both basic and instrumental activities of daily living (BADLs and IADLs, respectively) [5,6,7,8,9,10,11]. These abilities encompass essential self-care activities as well as more complex, goal-directed behaviors required for independent living, such as managing finances, medication adherence, social interactions, and household responsibilities. Functional decline in bvFTD is particularly devastating, as it reflects the loss of autonomy and is strongly associated with reduced quality of life, increased caregiver burden, earlier institutionalization, and substantial socioeconomic consequences, including loss of employment and increased healthcare utilization [5,6,7,8,9,10,11]. Therefore, functional status represents a clinically meaningful and highly ecologically valid outcome measure in dementia research, capturing the real-world disease impact more effectively than isolated cognitive test performance or global disease severity indices.

Importantly, bvFTD is often associated with a relatively rapid clinical course [5,9]. Longitudinal studies suggest that many patients progress to severe functional disability within approximately five years from symptom onset, although marked inter-individual variability in disease trajectories is consistently observed [5,6,7,8,9,10,11,12]. This heterogeneity highlights the need to identify clinically meaningful predictors of disease progression and functional decline, with the ultimate goal of improving prognostic accuracy and informing individualized clinical management [9,12].

Despite the clinical importance of functional outcomes in bvFTD, the number of longitudinal studies investigating predictors of functional decline remains limited [9,11,12,13,14,15,16,17,18,19]. Most existing research has relied on cross-sectional designs, examining associations between clinical features and functional impairment at a single time point rather than modeling change over time [5,6,7,8,20,21,22,23,24,25,26,27,28,29,30,31]. Even among longitudinal studies, follow-up intervals are heterogeneous and analytic approaches frequently focus on isolated predictors rather than integrative multivariate frameworks [7]. As a result, a comprehensive understanding of the multidimensional determinants of functional decline in bvFTD remains incomplete [12].

Functional impairment in bvFTD is thought to arise from the complex interplay of cognitive, behavioral, personality-related, and neurobiological factors [7,8]. From a cognitive perspective, global cognitive decline, executive dysfunction, and attentional deficits have consistently been associated with poorer everyday functioning [5,6,8,11,12,23,27,29,31]. Executive abilities are particularly relevant because they support planning, inhibitory control, cognitive flexibility, and goal-directed behavior, all of which are essential for independently managing daily activities [5,6,8,12,27,31]. Although episodic memory impairment may also be present in early-stage bvFTD [4], findings regarding its contribution to functional disability have been mixed [6,8,11,16,27]. Some studies have reported significant memory deficits and associations with everyday functioning [11,16,27], whereas others suggest that its contribution to functional disability may be secondary to impairment in executive control processes that support strategic retrieval and adaptive behavior, rather than primary memory storage deficits [8].

Social cognition represents another key determinant of functional outcomes in bvFTD [18,31]. Deficits in emotion recognition, theory of mind, sarcasm perception, and socioemotional semantic knowledge can significantly disrupt interpersonal interactions, social judgment, and adaptive behaviors in daily life [18,31].

Beyond cognitive and social cognitive deficits, behavioral disturbances represent a core feature of bvFTD and are strongly associated with functional decline [6,7,8,11,12,14,22,27,28,29,31]. In particular, negative behavioral symptoms, such as apathy, reduced initiative, emotional blunting, and loss of insight have consistently emerged as robust predictors of impaired everyday functioning [6,8,14,22,28,29,31]. Positive behavioral symptoms, including disinhibition and impulsivity, have also been linked to poorer functional outcomes, although findings are less consistent across studies [6,8,11,14,31].

Additionally, personality changes constitute a characteristic, yet comparatively underexplored, domain of bvFTD [1,2,3,8]. Alterations across personality traits, including conscientiousness, agreeableness, and emotional stability, have been consistently reported in patients with bvFTD and are thought to reflect early disruption of fronto-limbic and salience network systems [8,32,33,34]. Among these changes, reduced conscientiousness appears particularly relevant to everyday functioning due to its association with organization, planning, and goal-directed behavior [8,32,33,34].

Neuroimaging and neurophysiological studies further support the structural and functional basis of disability in bvFTD [8,12,15,17,21,25,26,34]. Atrophy and functional disruption in frontal and anterior temporal regions, particularly within right-lateralized networks, have been associated with more severe functional impairment and faster disease progression [12,26]. In addition, recent evidence has shown that regional brain hypoperfusion in prefrontal and parietal regions is closely linked to reduced daily functioning and greater disease severity [8,34]. Emerging evidence also suggests that neurophysiological and fluid biomarkers, such as measures derived from transcranial magnetic stimulation (TMS) and serum neurofilament light chain (NfL), may provide additional prognostic information regarding disease progression and functional decline in bvFTD [15,17].

### Study Aims

Although this body of research has significantly advanced understanding of bvFTD, important questions remain regarding the relative contribution of different disease domains to functional decline over time. The present study aimed to examine the multimodal correlates of longitudinal functional decline in bvFTD over a 12-month follow-up period. Specifically, through repeated comprehensive assessments conducted at baseline, 6 months, and 12 months, we sought to identify the cognitive, behavioral, personality-related, and neuroimaging factors most strongly associated with inter-individual variability in functional outcomes over time.

By integrating multiple domains of disease expression within a longitudinal framework, this study aimed to provide a more comprehensive characterization of the factors contributing to progressive functional impairment in bvFTD and therefore improve understanding of the marked heterogeneity observed in disease progression. The unique contribution of this work lies in its multimodal longitudinal design, combining clinical, neuropsychological, and neuroimaging assessments within the same analytical framework. This approach may help clarify the domains most relevant for prognostic stratification in bvFTD and provide a preliminary foundation for future research aimed at identifying individuals at increased risk of accelerated functional deterioration, thereby informing more individualized approaches to assessment, monitoring, and care.

## 2. Materials and Methods

### 2.1. Participants

This prospective longitudinal observational study included 27 patients who met the International Behavioral Variant FTD Consortium (FTDC) revised criteria [1] for at least possible bvFTD. Participants, together with their caregivers, were recruited from the 2nd Neurology Clinic of the “AHEPA” University General Hospital in Thessaloniki, Greece, between January 2023 and February 2025.

Diagnoses were established by a multidisciplinary team of neurologists and neuropsychologists following comprehensive clinical evaluation. As part of the diagnostic work-up, all participants underwent detailed neurological and neuropsychological assessment, structural and functional neuroimaging, genetic testing, and cerebrospinal fluid biomarker analysis to exclude alternative neurological or systemic causes.

Inclusion criteria required the availability of a knowledgeable informant (family member or close friend) capable of reliably reporting on the patient’s behavior, personality, and functional abilities. To focus on early-stage disease, only patients with symptom onset within the three years preceding study enrollment were included, based on informant report.

Exclusion criteria included the presence of significant vascular pathology on magnetic resonance imaging (MRI) or computed tomography (CT) that could confound neuroimaging interpretation, as well as any other neurological disorders (e.g., stroke, Parkinson’s disease, multiple sclerosis, epilepsy, or traumatic brain injury) capable of explaining cognitive or functional impairment. Participants with major psychiatric conditions (e.g., schizophrenia, bipolar disorder, or major depressive disorder) were excluded. Individuals with severe sensory, motor, or other physical impairments that could interfere with neuropsychological assessment of daily functioning were also not eligible.

### 2.2. Procedures

The study followed a longitudinal prospective observational design with repeated assessments conducted at three time points: baseline (T1), 6-month follow-up (T2; ±2 weeks), and 12-month follow-up (T3; ±2 weeks). Thus, participants were monitored over a total period of one year.

At each time point, participants were evaluated during scheduled outpatient visits at the memory clinic using standardized assessment protocols. Each assessment session lasted approximately 45 min and was conducted in quiet, well-lit rooms designed to minimize distractions and optimize cognitive performance. Testing was administered in the morning hours to reduce fatigue-related variability.

Caregivers were interviewed during scheduled clinic visits, typically across two structured sessions of approximately two hours each. Semi-structured interviews were conducted to obtain detailed information regarding patients’ behavioral changes, personality alterations, and functional abilities in everyday life, using validated informant-based instruments and disease staging scales.

All assessments were performed by certified clinical neuropsychologists with extensive experience in dementia research and clinical evaluation. A detailed description of all psychometric instruments is provided below.

Across all three time points, participants completed the full neuropsychological battery and informant-based questionnaires. Importantly, all cognitive, behavioral, personality, and functional measures were administered at each visit according to the study protocol. In contrast, brain perfusion SPECT imaging was acquired only at baseline (T1) and was not repeated at follow-up assessments.

The study protocol was approved by the Bioethics and Ethics Committee of Aristotle University of Thessaloniki (AUTh), Greece (protocol code: 331191/2022; approval date: 20 December 2022). Written informed consent was obtained from all participants and their informants. All data were anonymized in accordance with the European Union General Data Protection Regulation (GDPR; Regulation (EU) 2016/679) [35] and handled in full compliance with the Declaration of Helsinki [36].

### 2.3. Neuropsychological Assessment

A comprehensive neuropsychological battery [8] was administered to evaluate multiple cognitive domains, behavioral symptoms, personality changes, disease severity, and functional abilities.

Global cognitive functioning was assessed using the Montreal Cognitive Assessment (MoCA) [37], employing the psychometrically validated Greek adaptation [38]. Total scores (0–30) were used in the analyses, with higher scores indicating better cognitive performance.

The Clock Drawing Test (CDT) [39] was also administered as an additional measure of global cognition, using the validated Greek version [40,41]. Performance was rated on a 15-point scale, with higher scores reflecting better cognitive functioning.

Visuoconstructional abilities and episodic visual memory were evaluated using the Taylor Complex Figure Test [42]. Two indices were derived: (i) copy performance (0–36), reflecting visuospatial construction and perceptual organization, and (ii) delayed recall (0–36), indexing episodic visual memory.

Episodic verbal memory was assessed using two standardized tasks: the Word Learning Test and the Story Memory Test [41]. These evaluate memory for unrelated and semantically related materials, respectively. For both measures, only delayed recall scores were used (0–10 and 0–16), which were summed to generate a composite verbal memory index (0–26), with higher scores reflecting better performance.

Language abilities were assessed using the Confrontation Naming Test [41,43], which measures object naming and word retrieval. The total number of correctly named items (0–40) was used as the dependent variable, with higher scores indicating better naming performance.

Attention and psychomotor processing speed were evaluated using the Trail Making Test—Part A (TMT-A) [44], administered in its validated Greek version [45]. Performance was indexed by completion time, with shorter times reflecting better performance.

Short-term verbal memory and auditory attention were assessed using the Forward Digit Span Task [41,46], with scores reflecting the maximum number of digits correctly repeated in forward order (range: 0–9), where higher scores indicate better performance.

Executive functioning was assessed according to the widely accepted framework proposed by Miyake et al. (2000) [47], encompassing set-shifting, working memory updating, and inhibitory control.

Set-shifting was evaluated using the Greek version of the Trail Making Test—Part B (TMT-B) [44,45], with shorter completion times indicating better cognitive flexibility.

Working memory was assessed using the Backward Digit Span Task [41,46], with span scores ranging from 0 to 8, where higher values indicate better performance.

Inhibitory control was measured using the Stroop Test [48] in its validated Greek adaptation [49], which requires suppression of automatic reading responses in favor of color naming. The number of correctly identified ink colors was used as the outcome measure, with higher scores reflecting better inhibitory control.

Additionally, verbal fluency was assessed using the Verbal Fluency Test [46], in its validated Greek adaptation [50], including both semantic and phonemic conditions. Higher scores, defined as the total number of valid words generated, indicate better lexical retrieval and executive control.

A brief executive screening battery, the FRONTIER Executive Screen (FES) [51], was also administered. The Greek-adapted version [52] assesses phonemic fluency, verbal inhibitory control, and working memory. Each subtest is scored from 0 to 5, yielding a total score range of 0–15, with higher scores indicating better executive functioning.

In addition to conventional paper-and-pencil measures, computerized executive functioning was assessed using the REMEDES for Alzheimer-Revised (R4Alz-R) battery [53], a validated web-based neuropsychological assessment tool designed to evaluate attentional processes and executive control. For the purposes of the present study, only the total score of Part 3 of the battery was included in the analyses. This component assesses inhibitory control, task/rule switching, and cognitive flexibility. The score ranges from 0 to 179, with higher scores indicating poorer executive performance, corresponding to a greater number of errors and increased executive dysfunction.

Social cognition was evaluated using the Emotion Evaluation Test (EET), part of the Awareness of Social Inference Test—Short (TASIT-S) [54,55], assessing emotion recognition based on paralinguistic cues. Scores range from 0 to 10, with higher scores indicating better performance.

Theory-of-mind abilities were assessed using the Test of Social Inference—Minimal (SI-M), also part of TASIT-S [54,56], with scores ranging from 0 to 36; higher scores indicate better social inference ability.

Personality traits were assessed using the informant-rated Greek adaptation of the International Personality Item Pool (IPIP) Big Five questionnaire [57,58], based on the Five-Factor model of personality [59]. Given the well-documented impairment of insight in bvFTD [60,61,62], informant reports were used instead of patient self-reports. Ratings were provided in a third-person format, and composite scores (10–50) were computed for each of the five personality dimensions, with higher scores reflecting greater trait expression.

Behavioral symptoms were assessed using the Frontal Behavioral Inventory (FBI) [63], a validated 24-item informant-based scale specifically developed to quantify the presence and severity of behavioral symptoms typically associated with FTD [64]. The Greek version [65] was used in the present study. The FBI yields separate scores for negative symptoms (e.g., apathy, loss of insight) and positive symptoms (e.g., disinhibition, impulsivity), each ranging from 0 to 36, with higher scores indicating more severe behavioral disturbances.

Disease severity was evaluated using the Clinical Dementia Rating (CDR) scale [66], including both the standard version (CDR-GS; 0–3, CDR-SB; 0–18) and the FTLD-modified version (FTLD-CDR) [67,68,69], which incorporates additional language and behavioral domains (FTLD-CDR-SB; 0–24). Higher scores indicate greater disease severity.

The Greek version of the Frontotemporal Dementia Rating Scale (FRS) [19,68], a 30-item informant-based instrument, was additionally administered as an FTD-specific measure of disease staging, evaluating both behavioral disturbances and functional abilities. Raw FRS scores (maximum score = 30) were converted to percentage scores according to the standard scoring procedure, with higher percentages indicating less severe behavioral and functional impairment. The resulting percentage score was used in all subsequent analyses.

Functional status was assessed as the primary outcome using the Disability Assessment for Dementia (DAD) [70], in its validated Greek adaptation [6,8,71]. This informant-based scale evaluates both BADLs and IADLs, as well as initiation, planning/organization, and execution components. Scores are expressed as percentages, with higher values indicating greater functional independence. DAD was selected as the primary outcome measure due to its comprehensive assessment of both basic and instrumental daily functioning and its extensive validation in dementia and bvFTD research [5,6,7,8,14,22,23,28,29].

The Functional Activities Questionnaire (FAQ) [72] was also administered as an additional functional measure focusing primarily on IADLs, with higher scores indicating greater functional impairment.

### 2.4. Neuroimaging: Brain Perfusion SPECT

Regional cerebral blood flow (rCBF) was assessed at baseline using SPECT imaging at the 2nd Academic Nuclear Medicine Department of the “AHEPA” University General Hospital in Thessaloniki, Greece, in accordance with European Association of Nuclear Medicine (EANM) guidelines [73]. SPECT imaging was performed during the initial diagnostic assessment and was available only at the baseline visit; therefore, cerebral perfusion measures were considered baseline neuroimaging variables in the longitudinal analyses. Participants underwent imaging 30 min after intravenous administration of 740 MBq of technetium-99m-labeled hexamethylpropyleneamine oxime (^99m^Tc-HMPAO) at rest. Image acquisition was performed using a Philips gamma camera, and data were reconstructed using filtered back-projection techniques. Quantitative analysis of regional cerebral blood flow (rCBF) was performed using NeuroGam™ software (Segami Corporation, Columbia, SC, USA, www.segamicorp.com, accessed on 1 March 2026), with normalization to Talairach space and comparison against an age-matched normative database. For statistical analyses, z-score-transformed rCBF values were used to account for age-related variability in cerebral perfusion.

### 2.5. Statistical Analyses

#### 2.5.1. Preliminary Data Screening and Descriptive Statistics

All statistical analyses and data visualization were performed using the R statistical programming environment (version 4.5.3, www.r-project.org, accessed on 30 March 2026; R Foundation for Statistical Computing, Vienna, Austria) [74].

Prior to statistical modeling, descriptive statistics were computed to summarize participants’ baseline demographic and clinical characteristics. No attrition occurred during the follow-up period, and all 27 participants completed the full clinical, neuropsychological, behavioral, personality, and functional assessment battery at baseline, 6-month, and 12-month follow-up assessments. No missing data were present for longitudinal clinical assessments; therefore, no imputation procedures were required. The only exception concerned a single participant for whom SPECT imaging data were unavailable at baseline; this participant was excluded only from analyses involving neuroimaging variables.

#### 2.5.2. Longitudinal Changes Across the Follow-Up Period

Longitudinal changes in neuropsychological, behavioral, personality, functional, and disease severity measures across the 1-year follow-up period were evaluated using repeated-measures analyses of variance (ANOVA), implemented with the “ez” package in R. For each variable, time (baseline, 6-month follow-up, and 12-month follow-up) was specified as a within-subject factor. Repeated-measures ANOVA was used to assess whether performance changed significantly across the three assessment time points. To account for multiple comparisons, a Bonferroni correction was applied to the omnibus ANOVA tests by dividing the significance level (α = 0.05) by the total number of variables assessed (30 variables), resulting in an adjusted significance threshold of 0.0017. Statistical significance was then determined using this adjusted threshold to control the family-wise error rate (FWER) and reduce the risk of Type I errors.

For each analysis, *F*-statistics, associated *p*-values, and generalized eta-squared (η^2^_G_) effect sizes were extracted and reported. Assumptions of sphericity were evaluated using Mauchly’s test. When the assumption of sphericity was violated, Greenhouse–Geisser corrected *p*-values were used for statistical inference. For variables demonstrating a significant main effect of time, post hoc pairwise comparisons between assessment time points (baseline vs. 6 months, baseline vs. 12 months, and 6 months vs. 12 months) were conducted using Bonferroni-adjusted pairwise tests to identify the specific time intervals contributing to the overall effect. Means and standard deviations at each assessment time point were additionally computed to characterize patterns of longitudinal change.

#### 2.5.3. Predictor Screening and Dimension Reduction Strategy

For brain perfusion data, an initial neuroimaging-specific dimensionality reduction procedure was applied to limit the number of candidate SPECT variables and focus on regions demonstrating clinically meaningful abnormalities. Specifically, only BAs exhibiting hypoperfusion greater than 2 standard deviations below the normative mean in more than 50% of the sample were retained for subsequent analyses. This approach reduced the number of imaging variables included in the statistical models and ensured that analyses focused on regions most consistently affected within the cohort.

To identify the most informative predictors of functional decline while minimizing the risk of model overfitting, a two-stage screening and dimension reduction strategy was implemented prior to the primary longitudinal analyses.

First, bivariate Pearson correlation analyses were conducted between all candidate predictors and the primary functional outcome (DAD total score at 12-month follow-up). This preliminary screening step was used to prioritize variables showing significant evidence of association with subsequent functional status and to reduce the overall predictor space. Variables demonstrating significant associations at a threshold of *p* < 0.01 (**) were retained for further analysis. This initial screening step served as a first-stage dimensionality reduction procedure aimed at identifying the strongest correlates of functional impairment.

To further reduce dimensionality, variables surviving the preliminary correlation-based screening were entered into a Least Absolute Shrinkage and Selection Operator (LASSO) regression model using the “glmnet” package, with the alpha parameter set to 1 for L1 regularization. This method was selected due to its ability to perform simultaneous shrinkage and variable selection in the context of potentially correlated predictors, making it particularly suitable for high-dimensional datasets with limited sample size [75,76,77]. Before LASSO estimation, candidate predictors were standardized using the internal standardization procedure implemented in the “glmnet” package, ensuring that variables were placed on a comparable scale for penalized regression. The dataset was randomly divided into training (80%) and hold-out test (20%) subsets. LASSO model fitting was performed within the training subset, and the optimal penalty parameter (λ) was determined using 10-fold cross-validation, with λ__min_ selected as the value that minimized the cross-validated mean squared error (MSE). The resulting model was subsequently evaluated in the hold-out test subset to assess predictive performance. Nested cross-validation was not performed, as the primary objective of the LASSO procedure was predictor selection and dimensionality reduction prior to longitudinal mixed-effects modeling rather than the development of a fully optimized machine-learning prediction model. Furthermore, given the relatively small sample size, implementation of a nested cross-validation framework would have resulted in very small validation subsets and potentially unstable estimates of model performance.

The resulting LASSO model was subsequently evaluated using MSE and *R*^2^ to assess predictive performance. Predictors with non-zero coefficients were retained for inclusion in the subsequent longitudinal model. To further assess the potential optimism of the estimated model performance and evaluate internal validity, bootstrap-based validation was additionally performed. Bootstrap resampling was used to estimate the optimism in the apparent *R*^2^ of the final LASSO model by repeatedly refitting the model across bootstrap samples and comparing performance estimates between the bootstrap samples and the original dataset. The optimism-corrected *R*^2^ was calculated by subtracting the estimated optimism from the apparent model performance, providing a more conservative estimate of explained variance and accounting for potential overfitting associated with the small sample size.

This sequential two-step procedure, combining correlation-based screening with penalized regression-based variable selection, was implemented to reduce model complexity, mitigate the risk of overfitting, and identify the most informative candidate variables for inclusion in the subsequent longitudinal mixed-effects analyses.

#### 2.5.4. Linear Mixed-Effects Modeling of Longitudinal Functional Decline

The primary analysis of the present study consisted of linear mixed-effects (LME) modeling, conducted using the “lme4” and “lmerTest” packages in R. This approach was chosen to appropriately account for the repeated-measures structure of the data, in which repeated observations at baseline, 6-month, and 12-month follow-ups were nested within individuals. Importantly, all available measurements across all time points were included in the analysis through the use of a long-format dataset, thereby allowing full utilization of the longitudinal structure of the data.

LME models are particularly suited for longitudinal clinical data as they allow the simultaneous estimation of fixed effects, representing population-level associations between clinical variables and functional status, and random effects, capturing inter-individual variability in baseline performance and longitudinal change.

Maximum likelihood (ML) estimation was used for model fitting, allowing for the comparison of fixed effects within the model framework. Statistical inference for fixed effects was based on *t*-tests with Satterthwaite’s approximation for degrees of freedom, providing robust estimation of significance in the presence of repeated measures and complex variance structures.

Functional status (DAD total score) was specified as the dependent variable, with time included as a fixed effect to capture longitudinal change. Variables retained through the LASSO-based dimensionality reduction procedure were subsequently entered as fixed effects to examine their longitudinal associations with functional status across repeated assessments. Specifically, cognitive, behavioral, and personality measures were available at each assessment point and were incorporated as time-varying variables through the long-format dataset, whereas rCBF measures derived from SPECT imaging were available only at the baseline assessment and were therefore entered into the LME models as baseline predictors of longitudinal functional status. Time was also included as a fixed effect to model the overall trajectory of functional change across the follow-up period and to account for systematic within-subject changes over time. A random intercept for each participant was included to account for individual differences in baseline functional performance.

Unlike the LASSO procedure, variables entered into the LME models were not standardized and were retained in their original measurement scales. This approach was chosen to preserve the clinical interpretability of the estimated coefficients, allowing associations between individual clinical measures and functional status to be expressed in their original units.

Model fit was evaluated using Akaike’s Information Criterion (AIC), the Bayesian Information Criterion (BIC), and the log-likelihood statistic. The proportion of variance explained by the mixed-effects model was quantified using marginal and conditional *R*^2^ values, representing the variance explained by fixed effects alone and by the full model including both fixed and random effects, respectively.

Finally, to evaluate robustness and generalizability, the same analytical framework was applied using the FRS as a complementary outcome measure, commonly used to assess disease severity and functional status in bvFTD [11,13], thereby providing additional insights into the longitudinal correlates of functional abilities and disease severity in this clinical group.

## 3. Results

### 3.1. Demographic and Clinical Characteristics

The study sample comprised 27 patients with a diagnosis of bvFTD, including 12 males and 15 females. Participants had at baseline a mean age of 70.15 years (SD = 7.57), while the mean age at symptom onset was 68.04 years (SD = 7.70), indicating an average disease duration of approximately two years at study entry. The mean educational attainment was 10.85 years (SD = 3.90).

Regarding disease severity, the mean CDR-GS was 1.17 (SD = 0.67), and the mean CDR-SB score was 6.54 (SD = 3.81), findings consistent with mild-stage dementia [78]. As expected, the FTLD-modified CDR-SB score yielded higher values (M = 8.57, SD = 4.33), reflecting the additional contribution of behavioral and language impairments that characterize the FTLD spectrum.

Notably, two participants carried a genetically confirmed pathogenic mutation associated with bvFTD, specifically a C9orf72 repeat expansion. No known pathogenic FTD-related genetic mutations were identified in the remaining participants.

With respect to the knowledgeable informants who provided collateral information, spouses constituted the majority (63%), followed by adult children (30%) and siblings (7%).

Table 1 summarizes the baseline demographic and clinical characteristics of the study sample.

### 3.2. Longitudinal Changes in Neuropsychological, Behavioral, Personality, and Functional Measures Across the 1-Year Follow-Up Period

Descriptive statistics for all neuropsychological, behavioral, personality, disease severity, and functional measures across baseline, 6-month, and 12-month assessments, together with repeated-measures ANOVA results and Bonferroni-adjusted post hoc comparisons identifying the specific time intervals contributing to statistically significant longitudinal changes, are presented in Table 2.

Repeated-measures analyses revealed statistically significant longitudinal changes in most assessed variables, consistent with progressive and widespread disease-related deterioration over the 12-month follow-up period in bvFTD. Across cognitive, behavioral, personality, and functional domains, significant time effects indicated measurable changes over the course of disease progression. Bonferroni-adjusted post hoc comparisons demonstrated that longitudinal deterioration was most consistently observed between baseline and 12 months, while several measures also showed significant changes emerging as early as the 6-month assessment, indicating that decline was not restricted to the later stages of follow-up.

**Figure 1:**
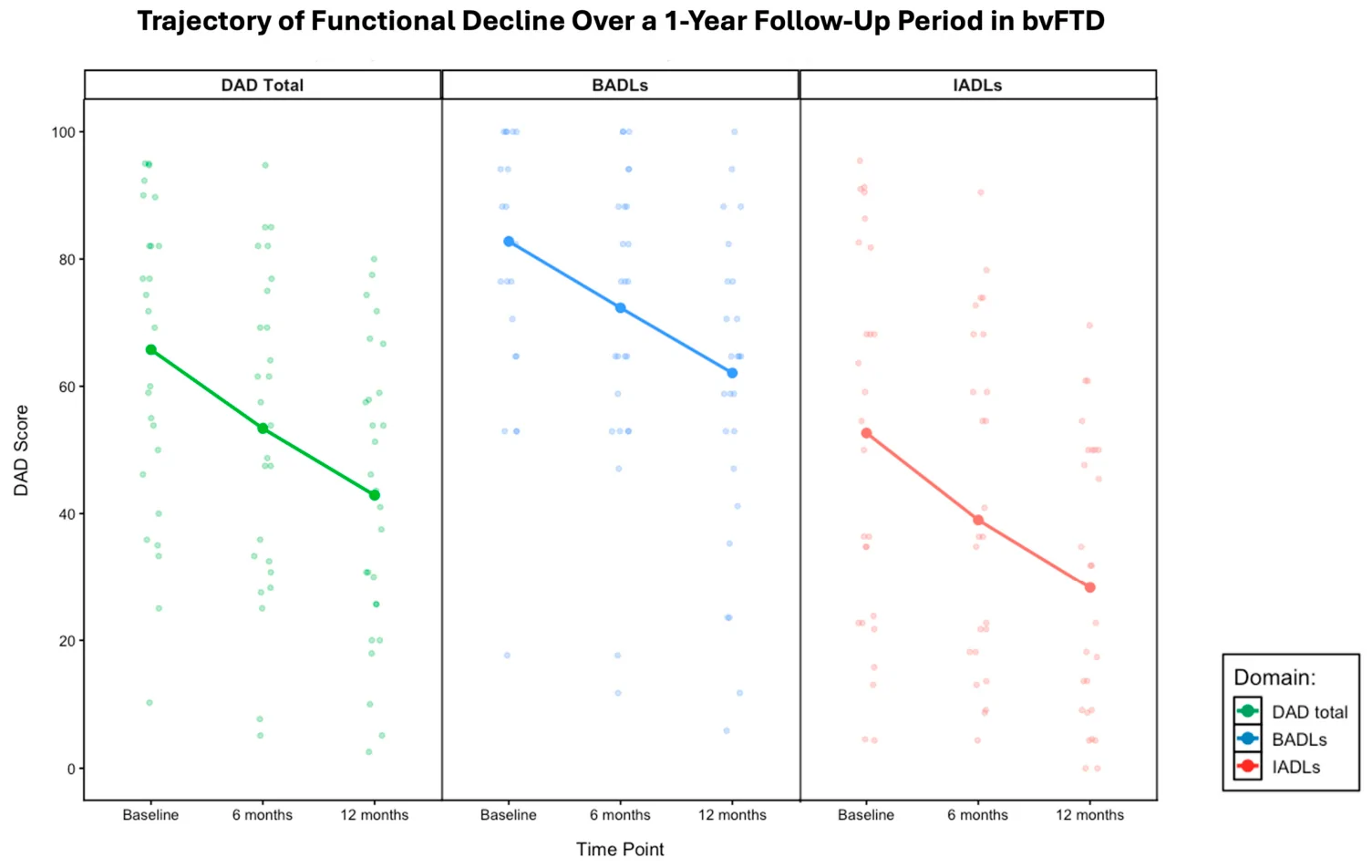
Progressive Functional Decline in bvFTD Over the 12-Month Follow-Up Based on DAD Total Score, as well as BADLs and IADLs sub-scores. Individual dots represent patient-level scores at each assessment point, while lines indicate the mean trajectory of functional change over time. Abbreviations (in alphabetical order): BADLs, basic activities of daily living; DAD, Disability Assessment for Dementia; IADLs, instrumental activities of daily living.

Regarding the primary outcome measure of the present study, the DAD scale demonstrated a substantial and progressive decline in functional abilities over the 12-month follow-up period (Figure 1). Total functional performance decreased markedly from baseline (65.76 ± 24.83) to 6 months (53.38 ± 24.35) and further to 12 months (42.89 ± 22.77), reflecting a clear worsening pattern in daily functioning over time (*F* = 28.39, *p* < 0.001, η^2^_G_ = 0.136). Bonferroni-adjusted post hoc comparisons confirmed significant deterioration across all assessment intervals (baseline–6 months, baseline–12 months, and 6–12 months), demonstrating a continuous decline in functional independence throughout the follow-up period.

A similar longitudinal pattern was observed when functional abilities were further decomposed into BADLs and IADLs. BADLs showed a significant and progressive decline across all time points (82.79 ± 20.95 at baseline, 72.33 ± 24.02 at 6 months, and 62.09 ± 26.28 at 12 months; *F* = 18.29, *p* < 0.001, η^2^_G_ = 0.115), indicating gradual deterioration in fundamental self-care abilities. Likewise, IADLs exhibited an even more pronounced decline over time (52.66 ± 30.74 at baseline, 38.99 ± 27.66 at 6 months, and 28.26 ± 22.40 at 12 months; *F* = 24.69, *p* < 0.001, η^2^_G_ = 0.123), reflecting a substantial loss of independence in more complex, cognitively demanding daily activities (Figure 1).

Overall, these findings indicate a robust and clinically meaningful decline in functional independence, with both BADLs and IADLs showing consistent deterioration across the 1-year follow-up period in bvFTD.

**Figure 2:**
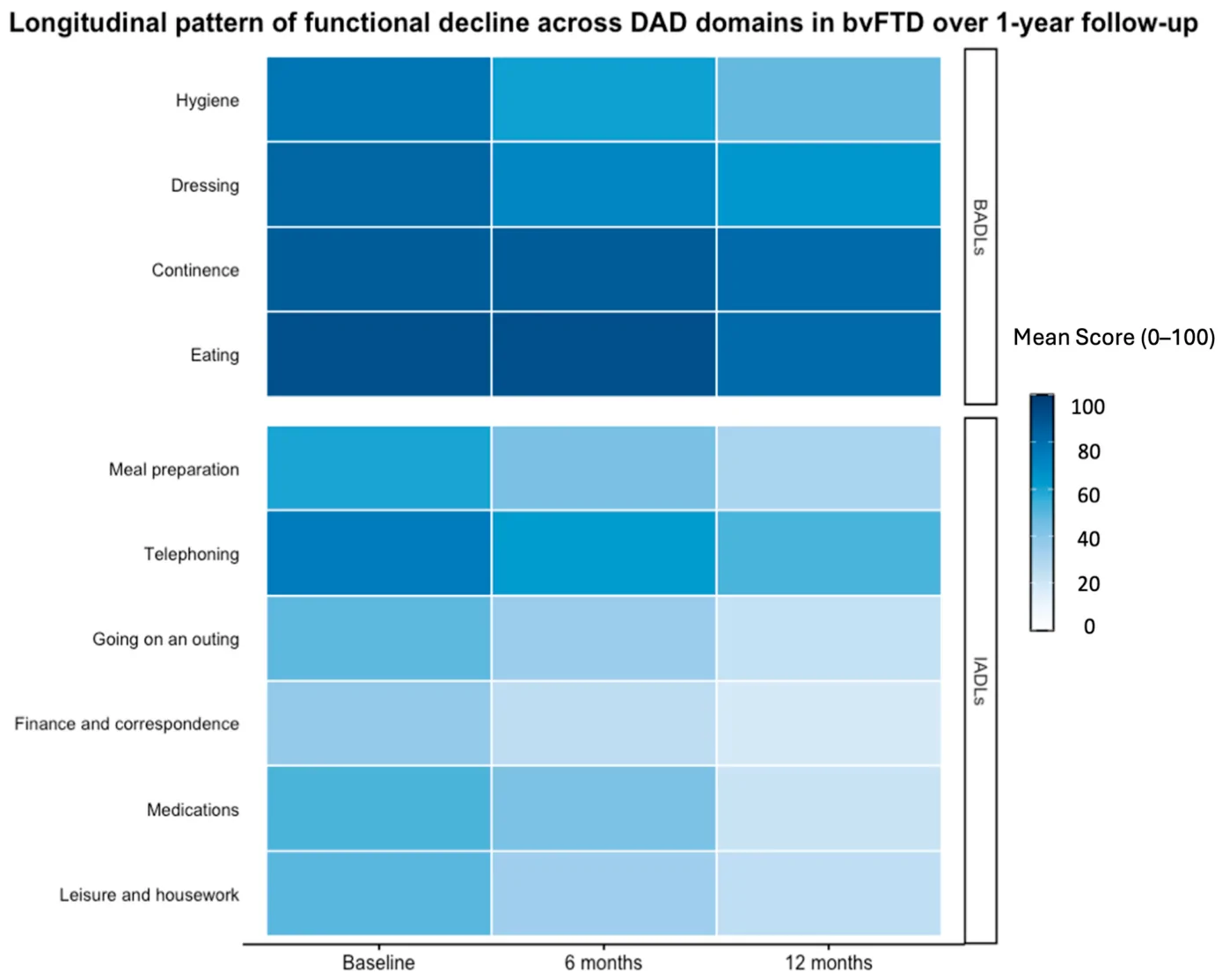
Domain-Specific Patterns of Functional Deterioration in bvFTD Over a 1-Year Follow-Up. Heatmap depicting mean DAD domain scores across baseline, 6-month, and 12-month assessments in individuals with bvFTD. Scores range from 0 to 100, with higher scores indicating better functional performance and greater preservation of independence. Color gradients represent mean domain scores at each time point, with darker blue shades indicating higher levels of preserved function and lighter shades reflecting greater functional impairment and loss of independence. Changes in color intensity across follow-up assessments illustrate longitudinal decline in domain-specific functional abilities. Domains are grouped into BADLs in the upper panel (hygiene, dressing, continence, and eating) and IADLs in the lower panel (meal preparation, telephoning, going on an outing, finance and correspondence, medications, and leisure and housework). Abbreviations (in alphabetical order): BADLs, basic activities of daily living; DAD, Disability Assessment for Dementia; IADLs, instrumental activities of daily living.

Additionally, Figure 2 illustrates the longitudinal pattern of functional impairment across the 10 individual DAD domains in bvFTD over the 12-month follow-up period. Changes in performance across BADLs and IADLs are visualized over time, highlighting domain-specific vulnerability and a progressive pattern of decline that appears earlier and is more pronounced in IADLs than in BADLs.

### 3.3. Preliminary Variable Screening and Penalized LASSO Regression Analysis

As outlined in the Methods section, an initial neuroimaging-specific dimensionality reduction procedure was applied to identify the most clinically relevant perfusion abnormalities. Of all measured Brodmann areas, only regions demonstrating hypoperfusion greater than 2 standard deviations below the normative mean in more than 50% of the sample were retained as candidate neuroimaging predictors. These regions included BA 5R, BA 6L, BA 8R, BA 9L, BA 11R, BA 20L, BA 24R, BA 28L, BA 31L, BA 32R, BA 37L, BA 40R, BA 44R, and BA 46R.

As an initial variable reduction step, Pearson’s correlation analyses were performed between all candidate predictors and DAD scores at the 12-month follow-up assessment. Of the variables examined, 14 met the predefined inclusion criterion of a statistically significant association with functional status (*p* < 0.01) and were therefore retained for further analyses.

The retained variables included the FBI-Negative Symptom sub-score, the IPIP Conscientiousness score, the MoCA total score, the Taylor Complex Figure copy score, the verbal memory composite score, the semantic verbal fluency performance, the completion time on the TMT-A, the confrontation naming performance, the Clock Drawing Test score, the Stroop Words and Colors condition score, the executive control composite score (Part 3) derived from the computerized R4Alz-R battery, the TASIT-S Part 2 total score, and the regional cerebral perfusion measures of right BA 8 and 24.

Prior to penalized regression modeling, multicollinearity among the selected predictors was assessed using Variance Inflation Factor (VIF) values. No evidence of problematic multicollinearity was observed, as all VIF values remained within acceptable limits.

The 14 retained variables were subsequently entered into the penalized LASSO regression model for further dimensionality reduction and predictor selection prior to longitudinal mixed-effects modeling. The optimal regularization parameter identified through the 10-fold cross-validation procedure was λ = 1.34 (λ__min_ = 1.340), corresponding to the minimum cross-validated prediction error. Using this level of penalization, the final LASSO model retained eight predictors with non-zero coefficients, indicating their contribution to explaining variability in DAD scores at the 12-month follow-up assessment.

Specifically, the retained predictors included the FBI-Negative Symptom sub-score (β = −1.04), the IPIP Conscientiousness score (β = 0.45), the MoCA total score (β = 1.33), the Taylor Complex Figure copy score (β = 0.60), the semantic verbal fluency performance (β = 0.49), the completion time on the TMT–Part A (β = −0.05), the Stroop Words and Colors score (β = 0.11), and the rCBF in right BA 24 (β = 1.03).

The intercept of the final model was estimated at 76.21. The remaining candidate predictors, namely the confrontation naming score, the verbal memory composite score, the Clock Drawing Test performance, the executive control composite score (Part 3) derived from the R4Alz-R battery, the TASIT-S Part 2 score, and the rCBF in right BA 8, were excluded from the final model as their coefficients were shrunk to zero by the LASSO penalization procedure.

The predictive performance of the model was acceptable, yielding a mean squared error (MSE) of 206.19 on the test dataset and explaining approximately 67.1% of the variance in DAD scores (*R*^2^ = 0.671). Given the small sample size and the potential for optimism in model performance estimates, bootstrap-based internal validation was additionally performed. This analysis estimated an optimism of 0.120, resulting in an optimism-corrected *R*^2^ of 0.551, indicating a more conservative estimate of the model’s explained variance after accounting for potential overfitting.

The variables retained through this two-step procedure were subsequently entered into the longitudinal LME model as candidate correlates of longitudinal functional decline.

### 3.4. Longitudinal Correlates of Functional Decline in bvFTD: LME Model Results

To identify the factors associated with functional status across the study period, a LME model was fitted with DAD scores as the dependent variable. The model incorporated repeated measurements obtained at baseline, 6-month, and 12-month follow-up assessments, while accounting for within-subject dependence through the inclusion of a participant-specific random intercept. The final model included time, as well as the eight variables retained following the previously described variable selection procedure. By utilizing a long-format dataset, the model was able to incorporate all available observations across all assessment waves, thereby fully leveraging the longitudinal structure of the data while accounting for the correlation among repeated measurements obtained from the same individual.

LME model fit indices indicated an acceptable fit to the observed data (AIC = 580.50, BIC = 608.80; log-likelihood = −278.20). The model explained 82.4% of the variance in functional status based on fixed effects alone (marginal *R*^2^ = 0.824), while the full model including both fixed effects and participant-specific random effects accounted for 95.1% of the variance (conditional *R*^2^ = 0.951).

Functional status showed a considerable decline over the follow-up period; however, the effect of time did not reach conventional statistical significance in the fully adjusted LME model (β = −0.43, SE = 0.22, *t* = −1.91, *p* = 0.061). Although borderline, the direction of the effect remained consistent with the expected progressive deterioration in functional abilities over time in bvFTD.

Five variables emerged as significant independent correlates of longitudinal functional status in the final LME model. At the behavioral level, greater severity of FBI-negative symptoms was associated with poorer functional outcomes, as reflected by lower DAD scores (β = −1.21, SE = 0.31, *t* = −3.87, *p* < 0.001). At the cognitive level, better global cognitive functioning, as measured by the MoCA (β = 1.51, SE = 0.43, *t* = 3.50, *p* < 0.001), faster processing speed and attentional efficiency, as assessed by the TMT–Part A (β = −0.18, SE = 0.03, *t* = −5.55, *p* < 0.001), and better inhibitory control abilities, as measured by the Stroop Words and Colors condition (β = 0.82, SE = 0.30, *t* = 2.78, *p* < 0.001), were all associated with higher levels of functional independence. At the neuroimaging level, greater rCBF within right BA 24 was also significantly associated with better functional status across repeated assessments (β = 0.79, SE = 0.24, *t* = 3.36, *p* < 0.001).

By contrast, semantic verbal fluency (β = 0.31, *p* = 0.527), Taylor Complex Figure copy performance (β = 0.35, *p* = 0.101), and the IPIP Conscientiousness personality trait (β = 0.002, *p* = 0.991) did not significantly predict functional status, after accounting for the effects of the remaining variables in the model.

The random-effects component of the model revealed substantial between-subject variability in baseline functional status (intercept variance = 91.65, SD = 9.57), supporting the presence of considerable inter-individual differences in functional performance among patients with bvFTD. The residual variance was estimated at 35.70 (SD = 5.98), indicating that a substantial proportion of variability in DAD scores was explained by differences between individuals rather than solely by within-person fluctuations over time.

The findings indicate that FBI-negative behavioral symptoms, global cognitive functioning (MoCA), attention and processing speed (TMT-Part A), inhibitory control abilities (Stroop-Words and Colors), and perfusion within right BA 24 represent the strongest independent longitudinal correlates of functional status in bvFTD. Importantly, these associations were identified while simultaneously accounting for repeated measurements across all three assessment waves and for individual differences in baseline functional performance.

### 3.5. Longitudinal Correlates of Functional Status Using the FRS as a Secondary Outcome Measure

To evaluate the robustness and generalizability of the findings obtained using the DAD as the primary functional outcome measure, the same analytical framework was applied using the FRS as a secondary measure of functional status.

Following the initial correlation screening procedure, twelve variables demonstrated significant associations with FRS scores (*p* < 0.01 **) and were retained for further analyses. These included FBI-Negative Symptoms, IPIP Conscientiousness, MoCA total score, Taylor Complex Figure copy performance, semantic verbal fluency, TMT-A completion time, Clock Drawing Test performance, Backward Digit Span, Stroop Words and Colors condition performance, the executive control composite score (Part 3) derived from the computerized R4Alz-R battery, the FES total score, and regional cerebral perfusion within right BA 8. These variables were subsequently entered into a penalized LASSO regression model. Using the optimal regularization parameter identified through 10-fold cross-validation (λ = 4.43), the final model retained five predictors with non-zero coefficients: FBI-Negative Symptoms (β = −1.26), MoCA (β = 0.71), Taylor Complex Figure copy performance (β = 0.05), the executive control composite score derived from the computerized R4Alz-R battery (β = −0.07), and regional cerebral perfusion within right BA 8 (β = 7.48). The model explained 55.9% of the variance in FRS scores (*R*^2^ = 0.559).

The retained variables were subsequently entered into a longitudinal LME model with FRS scores as the dependent variable. Consistent with the findings obtained using the DAD, a significant decline in functional status was observed over time (β = −0.92, SE = 0.25, *t* = −3.61, *p* < 0.001). At the behavioral level, greater severity of FBI-negative symptoms was associated with poorer functional outcomes, as reflected by lower FRS scores (β = −0.83, SE = 0.34, *t* = −2.47, *p* < 0.05). Additionally, among the retained predictors, better global cognitive functioning, as measured by the MoCA, was associated with better functional status across repeated assessments (β = 1.47, SE = 0.43, *t* = 3.39, *p* < 0.01). At the neuroimaging level, greater regional cerebral perfusion within right BA 8 was significantly associated with higher FRS scores (β = 7.32, SE = 2.37, *t* = 3.09, *p* < 0.01). By contrast, Taylor Complex Figure copy performance (β = 0.22, SE = 0.21, *t* = 1.06, *p* = 0.293) and the executive control composite score derived from the R4Alz-R battery (β = −0.07, SE = 0.08, *t* = −0.84, *p* = 0.403) did not emerge as significant independent predictors of functional status.

Model fit indices for the FRS-based LME model were as follows: AIC = 612.00, BIC = 633.20, and log-likelihood = −297.00. The model explained 67.8% of the variance in FRS scores based on fixed effects alone (marginal *R*^2^ = 0.678), while the full model including both fixed effects and participant-specific random effects accounted for 84.4% of the variance (conditional *R*^2^ = 0.844).

## 4. Discussion

The present study aimed to identify multimodal correlates of longitudinal functional decline in individuals with bvFTD by integrating behavioral, cognitive, personality, and neuroimaging measures within a prospective longitudinal framework. By examining repeated assessments over a 12-month period, we sought to characterize both the magnitude of functional change and the clinical and neurobiological factors associated with inter-individual variability in functional outcomes.

A first key finding was the marked and progressive deterioration in functional abilities over a relatively short follow-up period. Significant declines were evident across global functional status as well as across both BADLs and IADLs, indicating a widespread loss of independence over time in both basic and complex everyday activities [5,9]. Importantly, the deterioration was particularly pronounced in IADLs, which rely heavily on executive control, planning, organization, judgment, and self-monitoring capacities. This pattern is consistent with the clinical phenotype of bvFTD, in which impairments in executive and behavioral regulation frequently compromise complex daily activities before more fundamental self-care abilities become affected [6]. The observed trajectory aligns with prior longitudinal evidence suggesting a relatively aggressive course of functional deterioration in bvFTD compared with other neurodegenerative conditions, including AD and other FTD variants [5,7,9]. Even within a relatively brief 12-month window, participants experienced marked reductions in functional independence, a finding that likely carries profound implications for caregiver burden, supervision needs, healthcare utilization, and risk of institutionalization. These observations reinforce the importance of early identification of patients at heightened risk for accelerated functional decline and highlight the need for timely interventions aimed at preserving independence and optimizing long-term care planning.

Beyond documenting the extent of functional decline, an important observation of the present study concerns the range of factors associated with these longitudinal changes. Notably, the variables retained in the final longitudinal model were drawn from multiple domains, including behavioral symptoms, global cognition, attentional and executive functioning, and neuroimaging measures. This finding highlights the multifaceted nature of functional decline in bvFTD and suggests that loss of independence reflects the combined disruption of motivational, cognitive, and neural systems rather than impairment within any single domain [8,16]. The results therefore support a multidimensional conceptualization of disability in bvFTD and underscore the importance of multimodal assessment approaches that extend beyond isolated domains of impairment, when evaluating functional prognosis.

Among the examined predictors, FBI-negative behavioral symptoms [63] emerged as one of the strongest independent correlates of longitudinal functional status in bvFTD [6,8,14,21,28,29,31]. This finding is particularly noteworthy given that negative behavioral symptoms—including apathy, aspontaneity, emotional withdrawal, reduced initiative, indifference, and loss of motivation—represent core clinical features of the disease [1,2,3,6,7,79]. Unlike more fluctuating positive behavioral features, negative symptoms appear to reflect a relatively persistent reduction in behavioral activation systems that are essential for initiating and sustaining daily functioning [7,8,80]. Individuals who fail to initiate behavior, organize goal-directed actions, or maintain motivation are likely to experience functional impairment even when specific cognitive abilities remain relatively preserved [8]. Importantly, the present findings extend previous cross-sectional work in the field by demonstrating that negative behavioral symptoms are not merely concurrent correlates of disability but also relate to functional trajectories over time. These results reinforce their central role in the clinical expression of bvFTD and highlight their relevance for prognostic assessment.

Global cognitive functioning, as assessed by the MoCA [37], also emerged as a significant independent correlate of longitudinal functional status. Although bvFTD is often conceptualized primarily as a behavioral syndrome, accumulating evidence suggests that global cognitive impairment contributes substantially to disease progression and everyday functioning [7,8,10,11,29]. The MoCA provides a broad assessment of multiple cognitive domains, including executive functioning, attention, memory, language, visuospatial abilities, and orientation, thereby providing a global index of disease burden, probably more effectively than individual neuropsychological measures. Previous studies have similarly reported associations between global cognitive performance and functional outcomes in neurodegenerative disorders [7,8,10,11,29]. The present findings indicate that brief cognitive screening measures may offer clinically meaningful information regarding future functional trajectories, even in relatively early stages of the disease.

Attentional efficiency and processing speed, indexed by TMT-A performance [44], emerged as another robust correlate of longitudinal functional status. At first glance, TMT-A is often considered a relatively simple cognitive task compared with more complex executive measures. However, successful performance requires sustained attention, visual scanning, psychomotor speed, and efficient information processing, all of which constitute foundational cognitive processes supporting everyday functioning [8,44]. Everyday activities frequently require individuals to rapidly detect relevant information, sustain attention, monitor environmental demands, and respond appropriately to changing circumstances. Impairments in these basic attentional mechanisms may therefore have widespread downstream consequences for daily functioning. Notably, TMT-A had previously emerged as a significant correlate of functional status in our earlier cross-sectional work [8], and its continued significance in the present longitudinal analyses suggests that attentional efficiency may represent a stable and clinically relevant marker of both current and future functional capacity in bvFTD.

In addition, inhibitory control, as measured by the Stroop Words and Colors condition [48], was significantly associated with longitudinal functional outcomes. The Stroop paradigm is widely considered a measure of inhibitory control and the ability to suppress prepotent or automatic responses. Successful everyday functioning requires individuals to inhibit inappropriate or automatic behaviors, regulate impulses, resist environmental distractions, and flexibly adapt behavior according to situational demands. In bvFTD, breakdowns in these inhibitory mechanisms constitute hallmark features of the syndrome and contribute to socially inappropriate behavior and reduced self-regulation [1,2,3]. Poorer Stroop performance may therefore reflect a broader breakdown in executive control mechanisms that can directly contribute to loss of functional independence. Notably, the association between Stroop performance and functional status may also reflect aspects of behavioral disinhibition [81], including impulsive decision-making, tactless or inappropriate social comments, violations of social norms, and diminished restraint in interpersonal interactions, that overlap conceptually with positive behavioral symptoms.

At the neurobiological level, regional cerebral perfusion within right BA 24, corresponding to the ventral anterior cingulate cortex (ACC), emerged as a significant independent baseline predictor of functional status in bvFTD [82]. BA 24 constitutes a key subdivision of the ACC located along the medial frontal wall within the cingulate gyrus, a region that occupies a central position within large-scale frontal networks involved in motivation, error monitoring, conflict detection, behavioral regulation, goal-directed behavior, and cognitive control [82,83,84,85,86,87]. It is typically associated with the ventral and limbic-oriented sector of the cingulate cortex, which is strongly interconnected with orbitofrontal, insular, amygdalar, and striatal regions. Importantly, the anterior cingulate is also considered a key hub of the salience network, one of the neural systems most consistently implicated in bvFTD [82,83,84,85,86,87]. Dysfunction within this network has been consistently associated with apathy, impaired social behavior, reduced motivation, and executive dysfunction, all of which are key determinants of everyday functional disability in bvFTD [7,8]. Consistent with this network-based perspective, Alfano et al. (2022) [88] demonstrated that neuropsychiatric symptoms in patients with frontotemporal dementia are associated with alterations in large-scale functional connectivity patterns involving distributed neural systems implicated in affective and cognitive regulation. Using resting-state functional MRI, these authors identified abnormal connectivity patterns involving the default mode network, medial prefrontal cortex, and central executive network in patients with depression across FTD and Parkinson’s disease, suggesting that affective and behavioral disturbances in neurodegenerative disorders may arise from dysfunction within interconnected neural circuits rather than isolated regional abnormalities. These findings provide complementary support for the view that behavioral and neuropsychiatric manifestations of bvFTD reflect disruption of distributed frontal–cingulate and network-level systems. In this context, the association between right BA 24 perfusion and longitudinal functional status observed in the present study may reflect the integrity of broader neural circuits involved in motivation, emotional regulation, salience processing, and adaptive goal-directed behavior.

The present findings suggest that bvFTD individuals with relatively preserved perfusion within right BA 24 at baseline maintain better functional abilities over time, whereas reduced perfusion may reflect more advanced disruption of neural systems necessary for adaptive everyday behavior. These results further support the growing body of evidence indicating that neuroimaging biomarkers can provide meaningful prognostic information regarding disease progression and functional outcomes in bvFTD [12]. Additionally, the fact that perfusion within the right hemisphere emerged as predictive may be particularly noteworthy. Converging evidence suggests that right frontotemporal and medial frontal networks play a critical role in socioemotional processing, empathy, emotional regulation, and social conduct [8,34,89,90,91,92]. In neurodegenerative disorders, including bvFTD, degeneration of right-sided frontotemporal structures has been associated with loss of empathy and disturbances in interpersonal functioning [34,89,90,91,92], deficits that often have profound consequences for real-world independence and caregiver burden. Thus, preserved perfusion within right BA 24 may support the maintenance of socioemotional and behavioral capacities that are essential for adaptive daily functioning [18].

Interestingly, abnormalities involving BA 24 and adjacent anterior cingulate regions have also been reported in psychiatric disorders characterized by disturbances in emotion regulation, motivation, and behavioral control, including bipolar disorder and schizophrenia-spectrum conditions [86,93]. Although the etiopathogenic mechanisms underlying these disorders differ substantially from those of bvFTD, converging evidence suggests the involvement of partially overlapping neural circuits centered on the ACC and related salience-network regions. These observations are consistent with emerging transdiagnostic models proposing that psychiatric and neurodegenerative disorders, such as bvFTD, may share common vulnerabilities within large-scale networks subserving socioemotional processing, behavioral regulation, motivation, and executive control. Such convergence is perhaps not surprising given the prominent behavioral, affective, and neuropsychiatric disturbances that characterize bvFTD [1,2,3].

Interestingly, several variables that demonstrated significant associations during the initial screening procedures did not retain independent significance in the multivariate model. These included semantic verbal fluency, visuoconstructive abilities, and conscientiousness. Although variables were related to functional status at the univariate level, their effects were attenuated after accounting for the influence of other cognitive, behavioral, and neuroimaging predictors. This pattern may reflect substantial overlap between these measures and broader constructs such as overall cognitive status, executive-attentional efficiency, and behavioral dysregulation. Thus, while these measures appear to be important correlates of functional status [8], they may provide less unique explanatory value for longitudinal functional trajectories. For example, conscientiousness, while clinically relevant, may serve as a clinically informative marker of disease-related disruption, reflecting behavioral and executive dysfunction in bvFTD, rather than independently predicting longitudinal functional outcomes.

Equally noteworthy was the absence of verbal or visuospatial episodic memory measures among the final predictors. This finding is broadly consistent with the established neuropsychological profile of bvFTD, in which executive, behavioral, and social-cognitive disturbances typically play a more central role than episodic memory dysfunction, particularly during earlier disease stages [1,2,3]. Whereas memory impairment frequently represents a major determinant of functional decline in AD, functional deterioration in bvFTD appears to be driven primarily by disruptions in executive control, behavioral regulation, and motivational systems [7]. The present findings therefore provide additional support for the view that episodic memory deficits are not among the principal drivers of functional disability in bvFTD [8].

Similarly, social-cognitive measures and the computerized executive control measure derived from the R4Alz-R battery did not survive the final variable-selection procedures. One possible explanation is that many patients already exhibited substantial impairments in these domains at baseline, reducing their ability to differentiate future functional trajectories [8]. In other words, social-cognitive and complex executive deficits may be characteristic features of the syndrome itself, but once these impairments become widespread across patients, they may contribute less to prognostic discrimination. By contrast, measures such as TMT-A and Stroop performance may capture more subtle individual differences in attentional and inhibitory functioning that remain informative regarding subsequent functional outcomes.

An additional finding was the lack of independent association between FBI-positive symptoms and functional outcomes. Although symptoms such as disinhibition, impulsivity, and irritability are clinically prominent in bvFTD, their relationship with everyday functional impairment may be less direct than that of negative symptoms [7,8]. Positive behavioral features may also overlap with executive dysfunction measures, particularly inhibitory control tasks such as the Stroop test, which may partially account for their variance in multivariate analyses. In contrast, negative symptoms such as apathy and reduced initiative may exert a more direct impact on the initiation and maintenance of daily activities, thereby showing stronger associations with functional outcomes [7,8].

The secondary analysis using the FRS [19] as an alternative measure of functional status provided important convergent evidence supporting the robustness of the primary findings. Several variables associated with DAD performance also demonstrated significant relationships with FRS scores during the preliminary analyses, indicating substantial overlap in the factors underlying functional impairment across different measures of everyday functioning. Importantly, the final FRS model identified time, FBI-negative behavioral symptoms, and global cognitive functioning (MoCA) as independent correlates of longitudinal functional status, closely mirroring the pattern observed in the primary DAD analysis. The replication of these key behavioral and cognitive variables across two distinct functional outcome measures strengthens confidence in the stability and reliability of the observed associations and suggests that these factors represent core determinants of functional decline in bvFTD rather than measure-specific effects.

At the neurobiological level, rCBF within right BA 8 also demonstrated longitudinal prognostic value for FRS performance. BA 8 forms part of the dorsolateral prefrontal cortex and is implicated in higher-order executive processes, including attentional control, monitoring of environmental information, decision-making under uncertainty, working-memory operations, and the regulation of goal-directed behavior [8,34,94,95,96]. Although distinct from the ACC (BA 24) identified in the primary DAD analysis, BA 8 is extensively interconnected with broader frontal and cingulo-frontal networks that support executive control and adaptive behavior. Previous cross-sectional studies in bvFTD have linked dysfunction within this region to impaired functional status, behavioral disturbances, executive dysfunction and personality changes, and the present findings extend this literature by demonstrating its longitudinal predictive value [8,34].

Together with the DAD findings involving BA 24, the results suggest that the integrity of interconnected frontal–cingulate network systems may be particularly important for maintaining functional independence in bvFTD. While BA 24 may reflect motivational and salience-related mechanisms and BA 8 more closely reflects executive-attentional control processes [82,83,84,85,86,87,94,95,96], both regions contribute to the coordinated neural systems required for effective everyday functioning.

Overall, the substantial overlap observed across the two longitudinal models provide additional support for the robustness of the identified predictors, whereas the differences between models likely reflect the partially distinct functional domains and conceptual emphases captured by each outcome measure.

### 4.1. Clinical Implications

The clinical implications of these findings are substantial. Functional decline represents one of the most important outcomes in dementia because it directly determines patient autonomy, caregiver burden, quality of life, healthcare utilization, and institutionalization risk [97,98,99]. Identifying individuals who are at heightened risk for rapid functional deterioration is therefore of considerable clinical importance.

The present findings suggest that a relatively brief multimodal assessment battery incorporating behavioral measures (especially apathy-related symptoms), cognitive screening (MoCA), basic attentional measures (TMT-A), inhibitory control tasks (Stroop), and neuroimaging markers of frontal–cingulate network systems integrity may provide valuable information regarding functional status in bvFTD.

Such information may facilitate individualized care planning, improve prognostic counseling for patients and families, guide resource allocation, and assist clinicians in identifying patients who may require more intensive monitoring and support. Moreover, these markers may prove useful for patient stratification in future clinical trials targeting disease progression and functional outcomes [7].

### 4.2. Strengths and Limitations

Several strengths of the present study warrant mention. The longitudinal design enabled examination of factors associated with functional outcomes over time rather than merely cross-sectional associations. The use of repeated assessments allowed characterization of changes in functional abilities over the follow-up period, while the integration of behavioral, cognitive, personality, and neuroimaging measures provided a comprehensive multidimensional perspective. The combination of correlation screening, LASSO-based variable selection, and longitudinal mixed-effects modeling provided a structured analytical framework for reducing model complexity and identifying the most relevant longitudinal correlates within a relatively large set of candidate variables.

Nevertheless, several limitations should be acknowledged when interpreting the present findings. First, the sample size was small, reflecting the challenges inherent in recruiting and longitudinally following patients with relatively uncommon neurodegenerative syndromes such as bvFTD. Although the sample size is comparable to those reported in many longitudinal studies of bvFTD, it remains relatively limited in relation to the number of candidate predictors examined. Consequently, despite the use of a sequential variable reduction strategy involving correlation screening and penalized LASSO regression, the possibility of overfitting in the final LME model cannot be fully eliminated. The combination of a limited sample size and a relatively large predictor space may increase the risk of unstable parameter estimates and reduce generalizability of the identified associations. Accordingly, the findings should be interpreted with appropriate caution and considered preliminary until replicated and externally validated in larger independent cohorts.

An additional methodological consideration concerns the variable selection strategy employed prior to longitudinal LME modeling. Candidate variables were initially screened based on their associations with DAD scores at the 12-month follow-up assessment and were subsequently entered into a LASSO regression framework for dimensionality reduction. Although this data-driven but outcome-oriented approach was adopted as a pragmatic strategy to reduce model complexity and limit the number of variables entering the final longitudinal analyses, it may introduce an element of outcome-informed variable selection and potentially lead to optimistic estimates of the strength of the identified associations. To further evaluate the consistency of this approach, equivalent screening and LASSO procedures were additionally performed using baseline and 6-month DAD scores, which demonstrated substantial overlap in the retained variables. Nevertheless, these additional analyses cannot fully eliminate the possibility of bias introduced by outcome-informed variable selection.

In addition, the small sample size may have reduced statistical power, particularly for detecting small-to-moderate effects and more subtle associations among variables. As a result, potentially relevant variables may not have emerged as significant within the present analyses. Replication in larger multicenter studies is therefore warranted to establish the robustness, stability, and reproducibility of the identified predictors of functional decline.

Although the study incorporated a broad range of candidate predictors, additional biological markers, including structural MRI measures, genetic variables, and fluid biomarkers, were not examined and may contribute additional prognostic value. Furthermore, the follow-up period was limited to one year. Longer longitudinal investigations will be necessary to determine whether the identified predictors maintain their prognostic utility across later disease stages and over extended periods of disease progression.

An additional limitation concerns the interpretation of the neuroimaging findings. Although baseline right BA24 perfusion was identified as a significant predictor of longitudinal functional status, SPECT imaging was acquired only at study entry and was not repeated during follow-up. Therefore, the current design does not allow conclusions regarding how alterations in BA24 perfusion evolve over time or whether progressive changes in cerebral perfusion parallel functional deterioration. Future studies incorporating repeated neuroimaging assessments will be necessary to clarify the temporal relationship between neural changes and functional decline in bvFTD. In this context, recent longitudinal neuroimaging studies have demonstrated the value of repeated structural and diffusion MRI assessments for characterizing dynamic disease-related changes over time. For example, longitudinal MRI investigations have shown that accelerated brain atrophy, progressive microstructural white matter alterations, and temporally ordered biomarker changes may precede or accompany clinical deterioration and cognitive decline across extended follow-up periods [100,101,102]. Although these studies were conducted primarily in aging and Alzheimer’s disease cohorts, they highlight the potential of repeated multimodal neuroimaging assessments for capturing disease trajectories and identifying factors associated with differential progression rates. Similar longitudinal imaging frameworks may prove particularly valuable in bvFTD for tracking evolving neurodegenerative changes and elucidating how alterations within disease-relevant networks relate to functional deterioration and individual differences in clinical trajectories.

Future research should seek to validate the present findings in larger and more diverse cohorts, integrate multimodal predictors across cognitive, behavioral, neuroimaging, and biological domains, and develop clinically applicable prognostic models capable of accurately predicting individual functional trajectories. Such efforts may ultimately facilitate precision medicine approaches aimed at identifying patients at higher risk for accelerated decline and tailoring interventions accordingly [7,18].

Finally, future directions should also include comparative longitudinal investigations across dementia syndromes. Studies directly contrasting bvFTD with other neurodegenerative disorders, such as AD and other FTD phenotypes, may help to further delineate disease-specific patterns of functional decline. Such work would be instrumental in clarifying the unique clinical, cognitive, and neurobiological signatures of bvFTD, improving differential diagnosis, and enhancing our understanding of how distinct pathological processes translate into divergent trajectories of functional impairment.

## 5. Conclusions

Functional decline in bvFTD appears to be a complex and multifaceted process that reflects the interplay of behavioral, cognitive, and neurobiological alterations. Importantly, the marked decline in functional independence observed over just one year underscores the aggressive nature of functional deterioration that characterizes bvFTD and highlights its profound consequences for patient autonomy, everyday functioning, and the growing need for caregiver support. In the present study, apathy-related behavioral symptoms, global cognitive functioning, attentional efficiency, inhibitory control, and regional cerebral perfusion within networks involving the anterior cingulate and prefrontal cortex emerged as the most robust longitudinal correlates of functional status. Together, these findings suggest that loss of independence in bvFTD cannot be attributed to a single domain of impairment but is associated with the progressive disruption of multiple systems supporting motivation, salience processing, executive control, and adaptive everyday behavior. Understanding how these domains relate to functional outcomes may provide valuable insights into the mechanisms underlying disability and loss of independence in this population.

These findings underscore the importance of comprehensive multimodal assessments when evaluating prognosis and planning clinical management in bvFTD. Earlier identification of patients at greater risk for accelerated functional decline may facilitate individualized care planning, more accurate prognostic counseling, and improved targeting of supportive interventions. Ultimately, identifying reliable predictors of functional deterioration may contribute to the development of more personalized approaches to patient care, disease monitoring, and management in bvFTD, with the goal of improving clinical outcomes and quality of life for individuals affected by this complex and highly disabling syndrome.

Finally, although the present findings provide novel insights into the multidimensional contributors of longitudinal functional decline in bvFTD, this study should be considered exploratory and hypothesis-generating in nature. The identified associations provide a preliminary framework for understanding factors related to functional deterioration; however, replication and external validation in larger, independent, and preferably multicenter longitudinal cohorts will be essential to establish the robustness, generalizability, and potential clinical relevance of these findings before they can be translated into routine clinical practice.

## Figures and Tables

**Table 1:** Demographic and clinical characteristics of participants at baseline.

Mean (SD)	bvFTD Patients
**n**	27
**Age, years**	70.15 (7.57)
**Sex, M/F, n**	12/15
**Education, years**	10.85 (3.90)
**Age at symptom onset, years**	68.04 (7.70)
**Time since symptom onset, years**	2.07 (0.73)
**CDR Sum of Boxes**	6.54 (3.81)
**CDR Global Score**	1.17 (0.67)
**FTLD-CDR Sum of Boxes**	8.57 (4.33)

**Table 2:** Longitudinal Changes in Neuropsychological, Behavioral, Personality, Functional, and Disease Severity Measures Across a 1-Year Follow-Up Period in bvFTD.

Measure	Baseline Mean (SD)	6-Month Mean (SD)	12-Month Mean (SD)	*F*	*p*	*η* ^2^_G_	Post Hoc Comparisons
**FBI—Negative Symptoms**	19.41 (5.44)	23.19 (6.49)	27.07 (4.64)	47.42	<0.001 ***	0.247	BL–6M ***; BL–12M ***; 6M–12M ***
**FBI—Positive Symptoms**	13.19 (4.88)	15.26 (5.54)	17.00 (5.31)	12.09	<0.001 ***	0.084	BL–6M *; BL–12M ***
**IPIP—Conscientiousness**	22.67 (11.47)	22.00 (11.81)	17.26 (9.16)	10.47	<0.001 ***	0.048	BL–12M ***; 6M–12M **
**IPIP—Emotional Stability**	25.89 (11.61)	23.89 (11.98)	23.74 (13.83)	1.64	0.204	0.006	-
**IPIP—Intellect**	20.82 (7.20)	20.44 (8.07)	15.78 (5.48)	15.71	<0.001 ***	0.100	BL–12M ***; 6M–12M ***
**IPIP—Agreeableness**	29.19 (10.43)	28.67 (10.58)	23.59 (9.29)	13.04	<0.001 ***	0.061	BL–12M ***; 6M–12M ***
**IPIP—Extraversion**	24.74 (7.56)	25.56 (8.00)	21.07 (7.17)	6.61	<0.01	0.064	-
**MoCA**	15.11 (4.99)	13.30 (5.56)	10.82 (6.36)	22.25	<0.001 ***	0.091	BL–6M *; BL–12M ***; 6M–12M **
**CDT**	11.44 (3.63)	9.52 (3.56)	7.96 (4.21)	39.15	<0.001 ***	0.127	BL–6M ***; BL–12M ***; 6M–12M ***
**Taylor—Copy**	25.17 (8.94)	24.09 (10.22)	21.20 (11.53)	6.05	<0.01	0.027	-
**Taylor—Delayed Recall**	4.65 (5.19)	3.22 (4.39)	2.48 (3.92)	6.07	<0.01	0.039	-
**Verbal Memory—Delayed Recall**	4.04 (4.24)	3.11 (3.70)	2.33 (3.51)	14.05	<0.001 ***	0.033	BL–6M *; BL–12M ***
**Confrontation Naming**	33.22 (6.84)	30.48 (8.60)	27.89 (10.72)	10.76	<0.001 ***	0.059	BL–12M ***
**TMT—Part A**	120.93 (78.98)	134.26 (82.73)	157.19 (87.71)	11.11	<0.001 ***	0.032	BL–12M ***; 6M–12M *
**TMT—Part B**	247.70 (67.15)	256.56 (66.77)	267.00 (60.88)	5.37	<0.01	0.015	-
**Forward Digit Span**	5.52 (1.22)	4.89 (1.16)	4.37 (1.47)	20.96	<0.001 ***	0.121	BL–6M **; BL–12M ***; 6M–12M *
**Backward Digit Span**	3.59 (1.28)	3.15 (0.95)	2.93 (1.27)	5.10	<0.01	0.054	-
**Stroop—Words and Colors**	14.04 (7.53)	12.48 (8.19)	10.22 (7.07)	18.81	<0.001 ***	0.042	BL–6M *; BL–12M ***; 6M–12M **
**Semantic Verbal Fluency**	9.48 (3.90)	8.63 (4.51)	7.56 (4.69)	8.40	<0.001 ***	0.033	BL–12M ***
**Phonemic Verbal Fluency**	6.26 (3.25)	5.26 (3.68)	4.70 (3.80)	11.22	<0.001 ***	0.032	BL–6M *; BL–12M ***
**FES**	3.59 (3.20)	2.63 (2.57)	2.11 (2.26)	20.44	<0.001 ***	0.051	BL–6M ***; BL–12M ***
**R4Alz-R (Part 3)**	90.22 (27.35)	99.93 (33.30)	114.96 (33.77)	48.20	<0.001 ***	0.097	BL–6M **; BL–12M ***; 6M–12M ***
**TASIT-S EET**	5.48 (2.29)	4.70 (2.22)	3.85 (2.21)	16.05	<0.001 ***	0.084	BL–6M *; BL–12M ***; 6M–12M *
**TASIT-S SI-M**	23.30 (5.00)	22.07 (5.84)	18.89 (7.28)	18.18	<0.001 ***	0.087	BL–12M ***; 6M–12M ***
**DAD total**	65.76 (24.83)	53.38 (24.35)	42.89 (22.77)	28.39	<0.001 ***	0.136	BL–6M ***; BL–12M ***; 6M–12M **
**BADLs (DAD)**	82.79 (20.95)	72.33 (24.02)	62.09 (26.28)	18.29	<0.001 ***	0.115	BL–6M *; BL–12M ***; 6M–12M *
**IADLs (DAD)**	52.66 (30.74)	38.99 (27.66)	28.26 (22.40)	24.69	<0.001 ***	0.123	BL–6M ***; BL–12M ***; 6M–12M **
**FAQ**	16.48 (9.14)	20.78 (7.76)	24.82 (5.50)	26.57	<0.001 ***	0.171	BL–6M **; BL–12M ***; 6M–12M **
**FRS**	48.91 (22.37)	37.52 (20.91)	29.40 (18.08)	28.63	<0.001 ***	0.136	BL–6M ***; BL–12M ***; 6M–12M **
**FTLD-CDR Sum of Boxes**	8.57 (4.33)	11.91 (4.68)	15.39 (4.87)	61.00	<0.001 ***	0.273	BL–6M ***; BL–12M ***; 6M–12M ***

Longitudinal changes across time were examined using repeated-measures analysis of variance (ANOVA), with time (baseline, 6 months, 12 months) as the within-subject factor. The F statistic, associated *p* values, and effect sizes are reported. Effect sizes are expressed as generalized eta squared (η^2^_G_), which provides an appropriate measure of explained variance for repeated-measures designs. To account for multiple testing across the 30 repeated-measures ANOVAs performed, a corrected significance threshold of *p* < 0.0017 (0.05/30) was applied. Post hoc pairwise comparisons were conducted using Bonferroni-adjusted paired comparisons following significant repeated-measures ANOVA results (BL = baseline; 6M = 6-month follow-up; 12M = 12-month follow-up). Only statistically significant pairwise comparisons are reported in the Post hoc comparisons column. Levels of Statistical Significance: *p* < 0.05: *; *p* < 0.01: **; *p* < 0.001: ***. Abbreviations (in alphabetical order): BADLs, basic activities of daily living; BL = baseline; CDT, Clock Drawing Test; DAD, Disability Assessment for Dementia; FAQ, Functional Activities Questionnaire; FBI, Frontal Behavioral Inventory; FES, FRONTIER Executive Screen battery; FRS, Frontotemporal Dementia Rating Scale; IADLs, instrumental activities of daily living; IPIP, International Personality Item Pool; MoCA, Montreal Cognitive Assessment; R4Alz-R, REMEDES for Alzheimer-Revised battery (Part 3: computerized executive function measure assessing inhibitory control, task switching, and cognitive flexibility); TASIT-S EET, The Awareness of Social Inference Test—Short Form Emotion Evaluation Test; TASIT-S SI-M, The Awareness of Social Inference Test—Short Form Minimal Subtest; TMT, Trail Making Test; 6M = 6-month follow-up; 12M = 12-month follow-up.

## Data Availability

The raw data supporting the conclusions of this article will be made available by the authors on request.

## Abbreviations

The following abbreviations are used in this manuscript:

AD	Alzheimer’s Disease
ADLs	Activities of Daily Living
AIC	Akaike’s Information Criterion
BADLs	Basic Activities of Daily Living
BA	Brodmann Area
BIC	Bayesian Information Criterion
bvFTD	Behavioral Variant Frontotemporal Dementia
CDR	Clinical Dementia Rating
CDT	Clock Drawing Test
DAD	Disability Assessment for Dementia
FAQ	Functional Activities Questionnaire
FBI	Frontal Behavioral Inventory
FES	FRONTIER Executive Screen
FRS	Frontotemporal Dementia Rating Scale
FTD	Frontotemporal Dementia
FTLD-CDR	Frontotemporal Lobar Degeneration–Clinical Dementia Rating
IADLs	Instrumental Activities of Daily Living
IPIP	International Personality Item Pool
LASSO	Least Absolute Shrinkage and Selection Operator
LME	Linear Mixed-Effects
MAE	Mean Absolute Error
ML	Maximum Likelihood
MoCA	Montreal Cognitive Assessment
MSE	Mean Squared Error
NfL	neurofilament light chain
R^2^	Coefficient of Determination
R4Alz-R	REMEDES for Alzheimer-Revised Battery
rCBF	Regional Cerebral Blood Flow
SD	Standard Deviation
SPECT	Single-Photon Emission Computed Tomography
TASIT-S EET	The Awareness of Social Inference Test—Short Form, Emotion Evaluation Test
TASIT-S SI-M	The Awareness of Social Inference Test—Short Form, Minimal Subtest
TMS	transcranial magnetic stimulation
TMT-A	Trail Making Test Part A
TMT-B	Trail Making Test Part B
VIF	Variance Inflation Factor
η^2^_G_	Generalized Eta Squared
^99m^Tc-HMPAO	Hexamethyl Propylene Amine Oxime labeled with Technique-99m
